# Scaphoid Nonunion Arthroscopic‐Assisted Reduction and Percutaneous Fixation With Distal Radius Autologous Bone Graft

**DOI:** 10.1002/atn2.70048

**Published:** 2026-04-29

**Authors:** Anna L. Gorsky, Timothy J. Westbrooks, Kier M. Blevins, Krishna N. Chopra, Nina Suh, Eric R. Wagner

**Affiliations:** ^1^ Department of Orthopedics, Emory University Division of Hand, and Upper Extremity Surgery Atlanta Georgia U.S.A.

## Abstract

Scaphoid fractures are common and often progress to nonunion due to the unique vascularity. When nonunion occurs, patients are at risk of developing carpal collapse and arthritis if not managed appropriately with traditional surgical management. However, open surgical management puts the fragile vascular supply and extrinsic soft tissue stabilizers of the carpus at risk, compromising healing and clinical outcomes. Arthroscopy allows for the preservation of these structures and improved visualization of fracture debridement, reduction, and alignment. We describe an arthroscopic‐assisted reduction and percutaneous fixation of scaphoid nonunion with distal radius bone graft.

## Graphical abstract

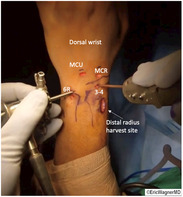

**VIDEO 1:** Arthroscopic assisted reduction and percutaneous fixation of scaphoid nonunion with autologous distal radius bone graft. Video demonstrating the use of arthroscopy in a scaphoid nonunion reduction. The fracture site is visualized in its entirety and debrided to bleeding bone before reduction is achieved. Bone graft is harvested from the ipsilateral distal radius and delivered to the nonunion site before and after percutaneous fixation with cannulated screws. Copyright Eric Wagner, M.D. Video content can be viewed at https://doi.org/10.1002/atn2.70048.

The scaphoid is the most commonly fractured carpal bone, accounting for 60%‐70% of all carpal fractures. It has been described as the “keystone” of the wrist, as it is the only carpal bone that bridges the proximal and distal carpal rows.^1^ This connection between the carpal rows is critical to maintain wrist biomechanics and prevent arthritic change.^2^

The scaphoid's predominant blood supply is via the dorsal carpal branch of the radial artery.^3^ The proximal pole also receives blood supply from the radioscapholunate ligament (ligament of Testut) and direct scapholunate branches from the palmar and dorsal transverse carpal arches.^4^, ^5^ Understanding and maintaining this vascular anatomy are critical to appropriately treating scaphoid fractures and nonunions.

Nonunion rates range from 5% to 15% with identifiable risk factors including smoking, interfragmental gap >1 mm, avascular necrosis of the proximal pole, and associated carpal instability.^6^, ^7^, ^8^, ^9^ In most cases, treatment consists of rigid internal fixation with vascularized or nonvascularized bone graft.^8^ Percutaneous, mini‐open, dorsal, volar, and arthroscopic approaches can be utilized depending on fracture location and pattern, as well as surgeon preference. For humpback deformity, most authors recommend a volar approach to allow correction of the intrascaphoid angle.^10^ Although open repair is an accepted treatment modality, arthroscopy has become more popular due to the preservation of vascular supply and extrinsic ligaments, for both waist and proximal pole nonunions.^11^, ^12^, ^13^

We describe an arthroscopic‐assisted reduction and percutaneous fixation of a scaphoid nonunion with distal radius autograft (Video 1). This technique maintains the carpal soft tissues to promote adequate healing, achieves stability using cannulated screws, offers a nonvascularized strut in which osteogenesis can occur, and can be utilized for both proximal pole and waist nonunions with or without humpback deformity.

## SURGICAL TECHNIQUE

### Position, Marking, and Portals

**FIGURE 1:**
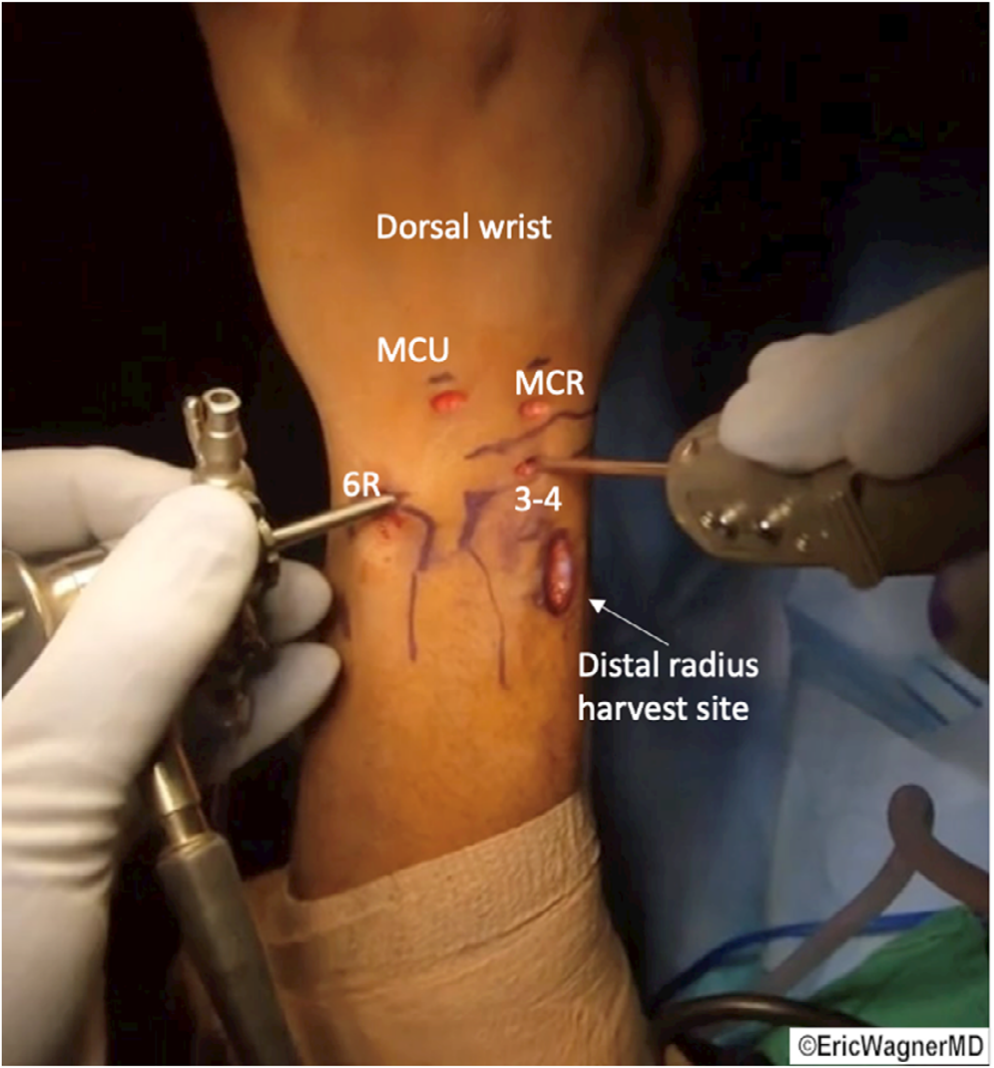
Arthroscopic portals for scaphoid nonunion reduction and percutaneous fixation. Illustration of dorsal wrist anatomy demonstrating the radiocarpal 3‐4, 6R, MCU, and MCR portals. The scope is in the 6R portal for viewing and the shaver is in the 3‐4 portal for debridement of the radiocarpal joint. For the midcarpal space, the MCU is the primary viewing portal and the MCR is the primary working portal. Copyright Eric Wagner, M.D. (6R, 6 radial; MCU, midcarpal ulnar; MCR, midcarpal radial.)

We use standard wrist arthroscopy portals including the 3‐4, 6 radial (6R), midcarpal ulnar, and midcarpal radial portals and perform this technique as a dry arthroscopy in the supine position and standard wrist traction tower (Figure 1). The graft harvest site is 1 cm proximal and radial to Lister's tubercle on the distal radius “bare spot.”

### Fracture Reduction

**FIGURE 2:**
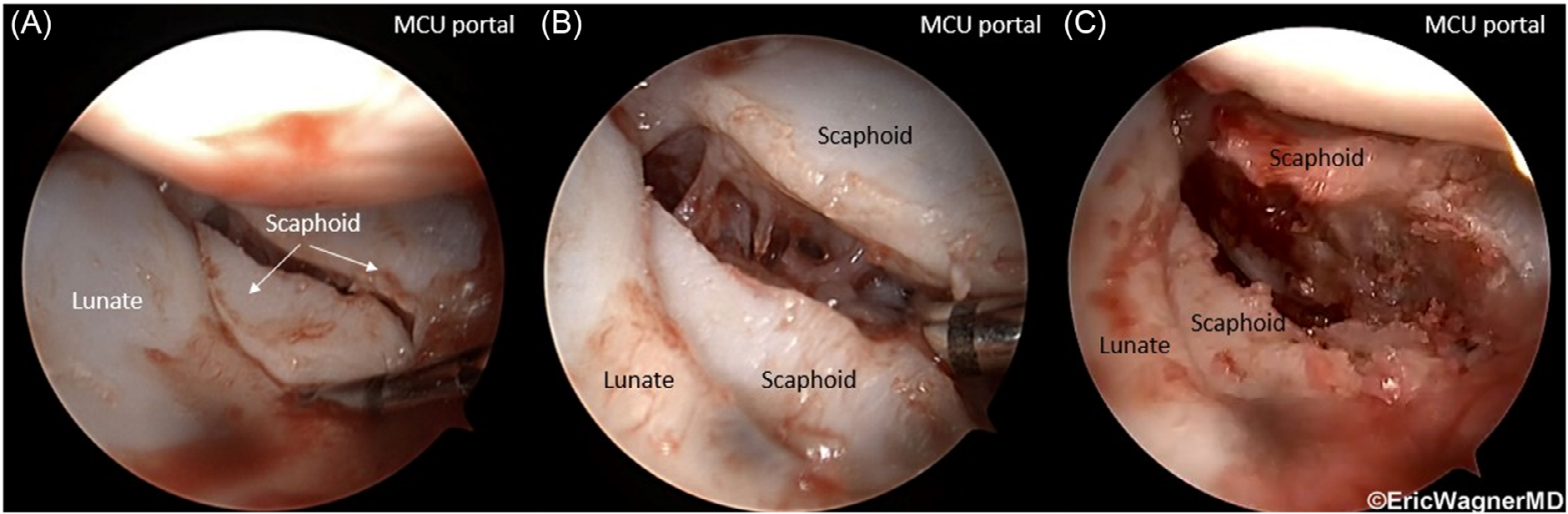
Visualization of the fracture nonunion site. (A,B) Two views of the scaphoid fracture nonunion site from the MCU portal. A probe examines the scapholunate ligament, lunotriquetral ligament, and nonunion site, and then a shaver and burr are sequentially introduced into the MCR portal to debride the nonunion site. (C) Viewing from the MCU portal, the nonunion site is debrided to bleeding bone using a shaver and burr. The fracture site is now ready for reduction. Copyright Eric Wagner, M.D. Left side; MCU viewing portal. (MCU, midcarpal ulnar; MCR, midcarpal radial.)

A standard diagnostic wrist arthroscopy is performed. The index and ring finger are placed in finger traps in 25‐30 pounds of traction. We create a 3‐4 portal, and then under visualization through the arthroscope, we establish a 6R portal. At this point, we can assess the radiocarpal joint, the volar carpal ligaments, radial styloid and proximal scaphoid articular cartilage, dorsal scaphoid translation, and proximal scaphoid. A motorized shaver debrides areas of inflammation and dorsal synovitis to aid in visualization. Full visualization of the proximal pole is necessary for fracture reduction and accurate screw placement (Table 1). The MCU portal is established and used as the viewing portal for the midcarpal space. The MCR portal is created in line with the scaphoid nonunion site. A probe is introduced to evaluate the scapholunate ligament, the lunotriquetral ligament, and the scaphoid nonunion site (Figure 2). The scaphoid nonunion site is debrided using a 3.0 mm arthroscopic shaver, followed by a 3.0 mm arthroscopic burr, all working through the MCR portal (Figure 2). Once the nonunion site is debrided back to healthy bleeding bone and all fibrous tissue is removed, it is ready for reduction and fixation.

**TABLE 1:** Pearls and Pitfalls

Pearls	Pitfalls
‐ Dry arthroscopy enables easier visualization for nonunion debridement and reduction, while facilitating graft placement and packing ‐ 25‐30 pounds of traction is used to allow appropriate distraction of the joint for ease of visualization and instrumentation ‐ The MCR portal should be made in line with the nonunion ‐ The 6R portal is used for visualization of the radiocarpal joint during wire and screw placement ‐ For proximal pole nonunions, after flexing the wrist 30°‐50° in a traction tower, the 3‐4 portal or a proximal and ulnar extension of it can be used to place the wire and screw ‐ For graft placement and packing, the MCU portal is the visualization portal and MCR is working portal ‐ A 3.5 mm drill sleeve or 2.9 mm arthroscopy canula can be used to shuttle the graft into the nonunion site ‐ If there is DISI deformity, a radiolunate pin should be placed first, then the humpback deformity reduced using wrist extension and ulnar deviation	‐ The third and fourth extensor compartments may obstruct the 3‐4 portal; careful retraction of these tendons away from the capsule is paramount to avoid damage during wire and screw placement ‐ Dorsal capsular synovitis should be debrided to allow for appropriate visualization for fracture reduction, implant placement, and autograft placement ‐ Failure to check the filling ratio after half of the bone graft is placed may prevent adequate reduction prior to screw placement ‐ If bone loss associated with the nonunion, a compression screw should be avoided, and instead use a noncompression screw or K‐wires ‐ A small amount of proximal bone in proximal pole nonunions, avoid a large 3.5 mm screw and instead use K‐wires or smaller‐diameter screws (1 or 2) ‐ To avoid malrotation of the fragment, use an antirotation K‐wire ‐ A SC and SL K‐wire is very helpful to maintain reduction but should not be placed until after the scaphoid fixation to avoid blocking the screw or midcarpal arthroscopic visualization

6R, 6 radial; DISI, dorsal intercalated segment instability; MCU, midcarpal ulnar; MCR, midcarpal radial; SC, scaphocapitate; SL, scapholunate.

**FIGURE 3:**
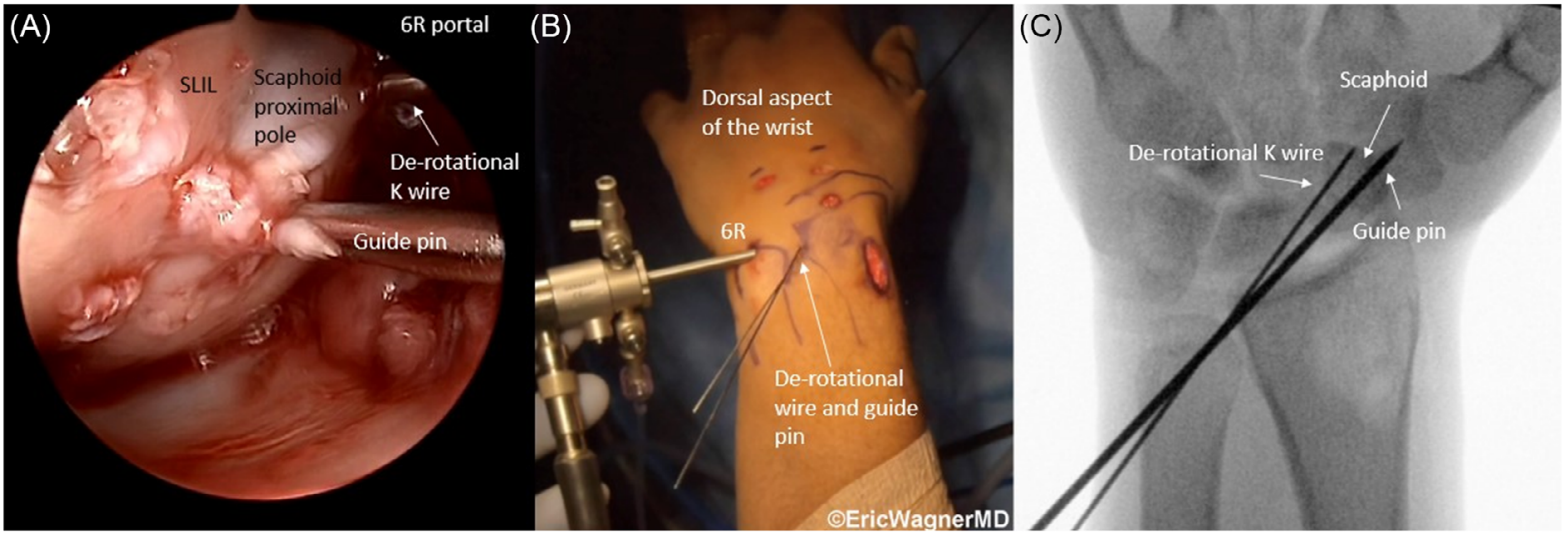
Reduction and percutaneous guide wire placement. (A) A derotational K‐wire is placed percutaneously into the proximal pole fragment, then across the nonunion site as seen in the top right corner of the arthroscopic image. The guide pin is then placed adjacent to the scapholunate ligament utilizing the derotational wire to reduce motion of the fragment as visualized through the 6R portal. (B) The wrist is flexed in the traction tower and the dorsal percutaneous pins are placed from an extended 3‐4 portal. The scope is in the 6R portal throughout the pin placement. (C) Intraoperative fluoroscopy of the wrist demonstrates the guide pin and derotational K‐wire traversing the 2 fracture segments of the scaphoid after reduction. Once fluoroscopy confirms the reduction and acceptable pin placement, the bone graft is harvested. Copyright Eric Wagner, M.D. Left side; 6R viewing portal. (6R, 6 radial; SLIL, scapholunate interosseous ligament.)

The reduction and guidewire placement depend on the location of the fracture and if a humpback deformity is present. If there is a humpback, the fracture is reduced via the “Linscheid maneuver,” extending the wrist in ulnar deviation over a stack of folded towels and inserting the guidewire via the volar approach in a center‐center position. In cases of proximal pole nonunions or stable waist nonunions without humpback malignment, a dorsal arthroscopic‐assisted approach is used (Figure 3). The wrist is flexed in the arthroscopy tower and reduction of the scaphoid is confirmed with fluoroscopy (Figure 3). The 6R portal is used for visualization, while the 3‐4 portal is extended ulnarly and proximally to enable guidewire insertion. Extensor tendons are carefully retracted away from the exposed capsule in the 3‐4 portal. Under arthroscopic visualization, a 0.045‐inch smooth derotation Kirshner wire is placed percutaneously through the 3‐4 portal into the proximal pole fragment, then across the nonunion site. A central guide pin is placed percutaneously through this dorsal approach, adjacent to the scapholunate ligament. If 2 screws are utilized, one Kirshner wire is placed in a more volar position and the other in a more dorsal position. Once the guidewire position and reduction are confirmed on multiplanar fluoroscopy, the bone graft is harvested. The guidewires are then advanced out volarly to allow the wrist to be placed back into traction.

### Bone Grafting and Percutaneous Fixation

**FIGURE 4:**
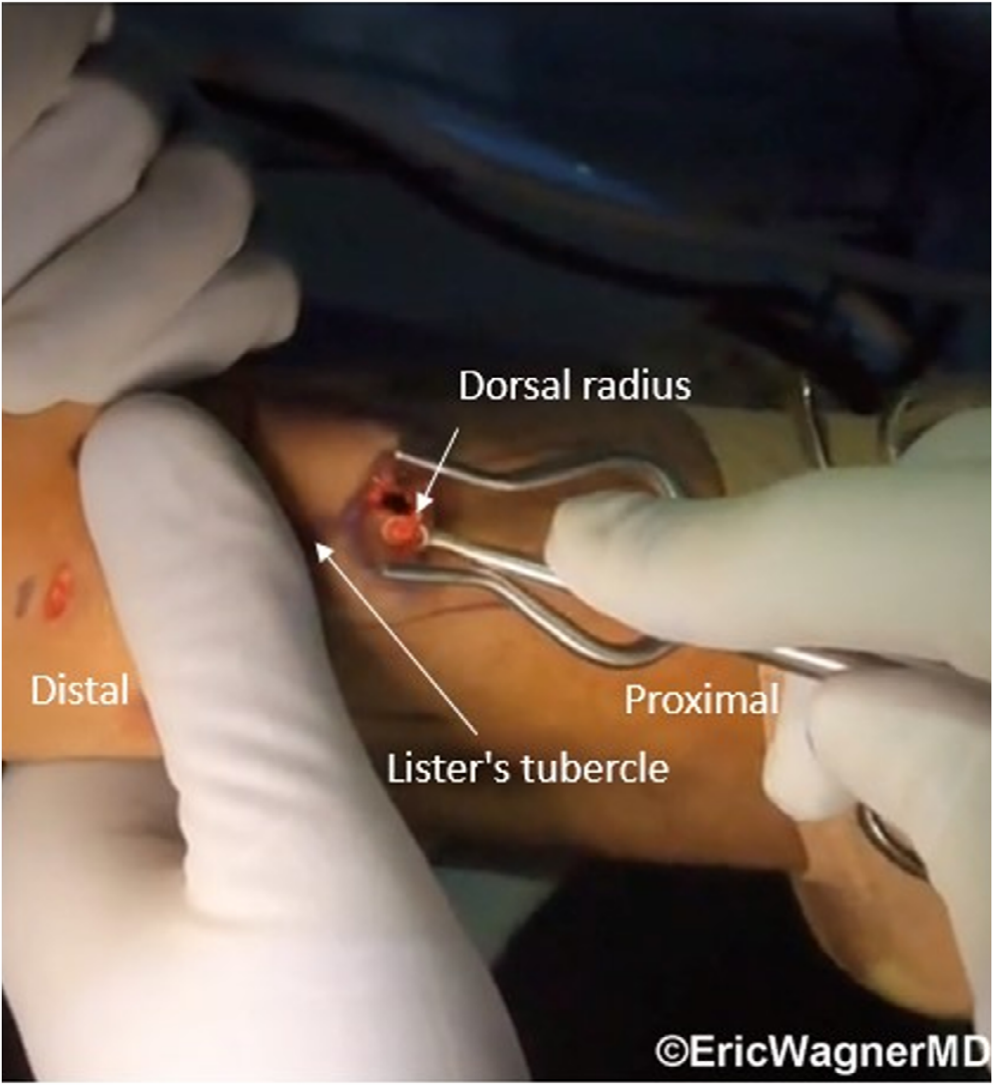
Distal radius autologous bone graft harvest. Cancellous bone graft is harvested from the ipsilateral distal radius via a dorsal longitudinal incision in the bare area of the radius, 1 cm radial and proximal to Lister's tubercle using the Acumed Bone Graft Harvesting System (Hillsboro, OR) and a curette. The bone graft is saved until screw fixation. Copyright Eric Wagner, M.D.

**FIGURE 5:**
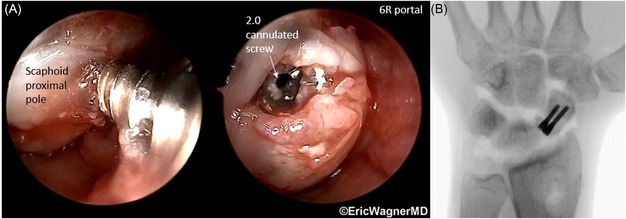
Fixation with 2.0 mm cannulated screws. (A) Viewing the scaphoid proximal pole from the 6R portal, the drill is advanced for placement of the cannulated screws. We utilized both the derotational K‐wire site and central K‐wire to place two 2.0 mm cannulated screws. If there is less proximal bone available, one 3.5 mm cannulated screw is adequate. (B) Intraoperative fluoroscopy of the wrist demonstrating 2 screws crossing the nonunion site. When 2 screws are utilized, one screw is placed more dorsal, and the other is more volar. Copyright Eric Wagner, M.D. Left side; 6R viewing portal. (6R, 6 radial.)

**FIGURE 6:**
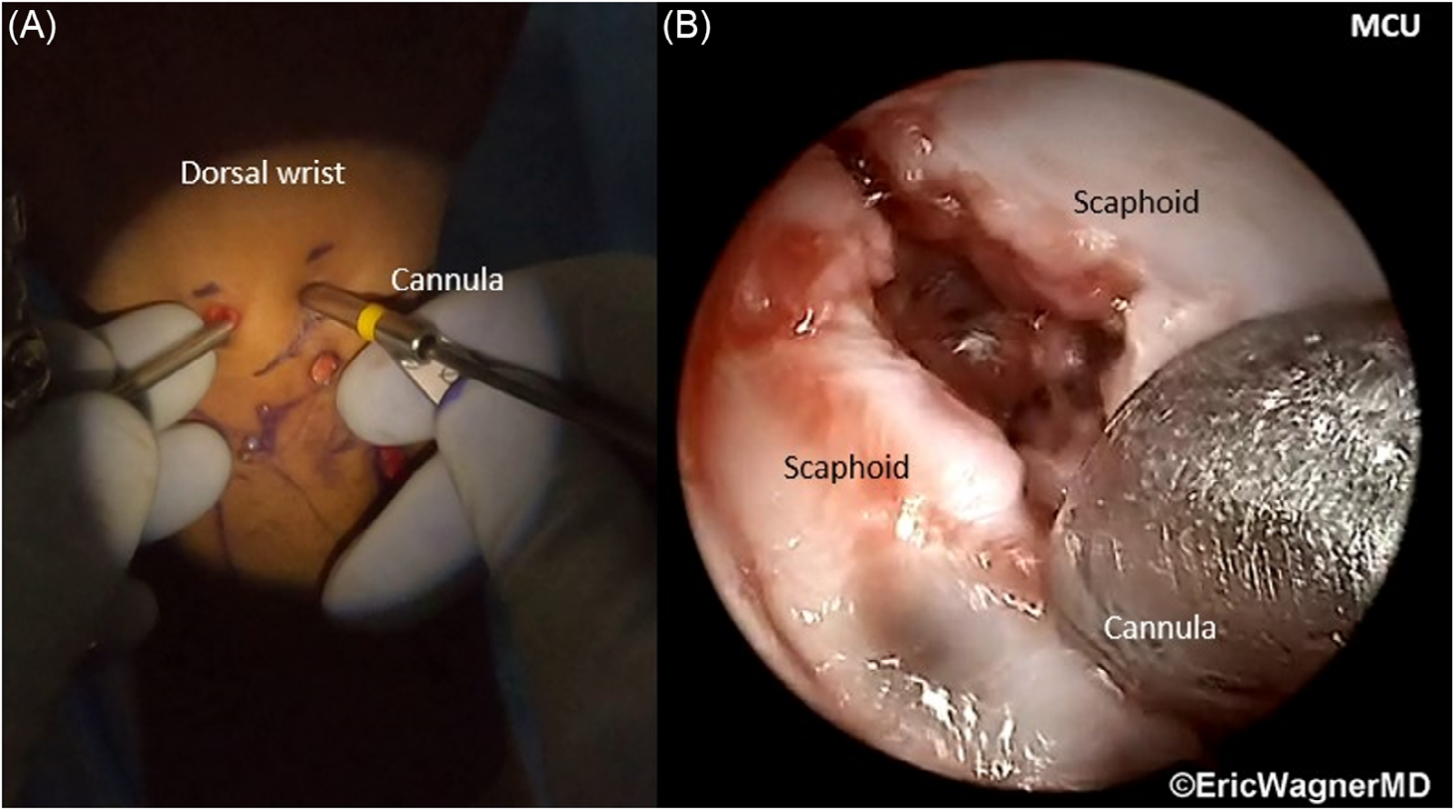
Delivering distal radius bone graft. (A) Bone graft is delivered into the nonunion site via a 3.5 mm cannula or drill sleeve in the MCR portal on the dorsal aspect of the wrist. (B) Viewing through the MCU portal, the cannula in the MCR portal is positioned over the nonunion site and bone graft is delivered. Once half of the bone graft is delivered, the filling ratio is checked before proceeding. Copyright Eric Wagner, M.D. Left side; MCU viewing portal. (MCR, midcarpal ulnar; MCU, midcarpal radial.)

**FIGURE 7:**
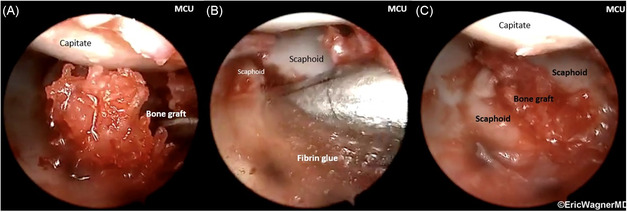
Packing distal radius bone graft. (A) The remaining half of the distal radius bone graft is inserted through the MCR portal while viewing from the MCU portal. The graft is packed down to fill the remaining space after scaphoid fixation. (B) Fibrin glue is inserted through the MCR portal via a cannula to secure the autograft in place. (C) The final construct viewed through the MCU portal with distal radius autograft secured with fibrin glue and packed down between the fixed fragments of the scaphoid. Copyright Eric Wagner, M.D. Left side; MCU viewing portal. (MCR, midcarpal ulnar; MCU, midcarpal radial.)

A small longitudinal incision is made 1 cm proximal and radial to Lister's tubercle in the “bare spot” of the distal radius (Figure 4). Cancellous bone graft is harvested using the Acumed Bone Graft Harvesting System (Hillsboro, OR). A 3.5 mm cannula or drill sleeve is then used to deliver the bone graft through the MCR portal into the nonunion site. About half of the space is filled before the filling ratio is checked. A percutaneous depth gauge is then placed over the Kirshner wires to measure the appropriate length cannulated screw(s). We typically utilize either one Acutrack 2 Mini (Hillsboro, OR) 3.5 mm cannulated screw or two Acutrack 2 Micro (Hillsboro, OR) 2.5 mm cannulated screws. Fluoroscopy and direct arthroscopic visualization confirm final screw position and depth, ensuring that the screws are deep to the chondral surface (Figure 5). The remainder of the autograft is packed until the entire area of nonunion is filled (Figure 6). Fibrin glue is placed to seal the graft in place (Figure 7). Finally, biplanar fluoroscopy and midcarpal arthroscopy confirm the placement of the graft and hardware (Figure 7).

### Postoperative Management

Patients are immobilized in a thumb spica splint for 14 days, transitioning to a removable thumb spica for 6‐8 weeks. If >30% healing is seen on computed tomography at 6 weeks, the patient is transitioned out of the splint. Active/passive motion and strengthening begins, with full activity resumed at 16 weeks.

## DISCUSSION

Scaphoid fractures are the most common carpal fracture and can have devastating outcomes if not adequately addressed. Even if treated appropriately, a significant percentage of fractures progress to nonunion.^6^, ^7^, ^8^ The unique vascularization of the scaphoid contributes to the challenging pathology and is exacerbated by some nonmodifiable risk factors such as the fracture site location, displacement of the fragments, and the presence of ligamentous injury.^9^

Our technique allows for the anatomic reduction of the scaphoid under direct visualization while maintaining critical soft tissue structures (Table 2). Malalignment of the fracture or disruption of the carpal ligaments can lead to negative functional outcomes.^14^, ^15^, ^16^ Studies have shown that the use of arthroscopy and bone grafting can restore wrist function and reduce pain in these challenging cases.^13^, ^17^, ^18^, ^19^ A 2024 systematic review found that union rates ranged from 86% to 100% in a time frame of 2.3 to 7.8 months, clinical outcomes were satisfactory, and complication rates were low when scaphoid nonunions were treated with arthroscopy.^20^ Oh et al. performed a comparative analysis of open versus arthroscopic treatment of scaphoid nonunions and found no significant differences in union rates, clinical scores, or radiographic assessments at 2 years postoperatively.^21^ Additionally, arthroscopy allows the surgeon to evaluate for other radiocarpal and midcarpal injuries, clearly visualize the presence of arthritis, assist with and visualize scaphoid reduction, precisely place screws in proximal fragments, and evaluate reduction and stability of the fragments after screw placement.

**TABLE 2:** Advantages and Disadvantages

Advantages	Disadvantages
‐ Arthroscopy allows for clear and direct visualization of the fracture, facilitating anatomic reduction and graft placement ‐ Arthroscopy protects the critical dorsal and volar carpal ligaments and the scaphoid vascular supply ‐ Arthroscopic visualization allows for a thorough debridement under direct visualization back to bleeding bone ‐ Nonvascularized distal radius autograft allows for minimal donor site morbidity ‐ Arthroscopy enables visualization of the reduction after fixation, as well as final graft positioning ‐ Arthroscopy allows for identification and treatment of associated intra‐articular pathology ‐ This technique can be utilized for different fracture patterns, including in cases of humpback deformity. It also enables insertion of the wires/screw dorsally, volarly, or in a combined fashion	‐ Arthroscopy for scaphoid nonunion requires proficiency of wrist arthroscopy, which may present a learning curve for surgeons who have traditionally performed these cases via the open approach ‐ Arthroscopy for scaphoid nonunions is technically challenging, likely taking more time that many open techniques ‐ It is not possible to perform a vascularized structural bone graft using arthroscopy

The use of bone graft in scaphoid nonunion is common, but there is not a clearly preferred choice of graft or method of harvest in the current literature. While some studies demonstrate superior union rates and faster time to union with vascularized bone grafts, vascularized bone grafts are typically reserved for cases of avascular necrosis due to the technical complexity and donor site morbidity.^22^, ^23^ Our technique is consistent with existing literature demonstrating no significant difference in union and functional outcomes with the use of nonvascularized bone graft.^24^ Pinder et al. reported no significant differences between vascularized and nonvascularized grafts^8^ and Lin et al. found that nonvascularized graft provided satisfactory results even for patients with avascular necrosis of the scaphoid.^19^ Furthermore, the arthroscopic approach optimizes intrinsic vascularity, further supporting the choice of nonvascularized graft.

Scaphoid nonunion presents a considerable challenge and lacks a standard treatment. This arthroscopic technique with distal radius bone graft protects the fragile anatomy of the carpal soft tissue and offers better visualization while also providing satisfactory radiographic and clinical results.

## DISCLOSURES

The author (E.R.W.) declares the following financial interests/personal relationships, which may be considered as potential competing interests: E.R.W. reports a relationship with Stryker and Acumed LLC that includes: consulting or advisory and travel reimbursement; reports a relationship with Smith & Nephew and DePuy Synthes that includes: consulting or advisory; reports a relationship with Konica Minolta that includes: funding grants; reports a relationship with Arthrex, Wright Medical Group, and Integra LifeSciences Corporation that includes: travel reimbursement. The other authors (A.L.G., T.J.W., K.M.B., K.N.C., N.S.) declare that they have no known competing financial interests or personal relationships that could have appeared to influence the work reported in this paper.
